# Call Characteristics of Patients Suspected of Transient Ischemic Attack (TIA) or Stroke During Out-of-Hours Service: A Comparison Between Men and Women

**DOI:** 10.3389/fneur.2021.669090

**Published:** 2021-06-14

**Authors:** Lieza G. Exalto, Sander van Doorn, D. Carmen A. Erkelens, Karin Smit, Frans H. Rutten, L. Jaap Kappelle, Dorien L. M. Zwart

**Affiliations:** ^1^Department of Neurology and Neurosurgery, UMC Utrecht Brain Center, University Medical Center Utrecht, Utrecht University, Utrecht, Netherlands; ^2^Department of General Practice, Julius Center for Health Sciences and Primary Care, University Medical Center Utrecht, Utrecht University, Utrecht, Netherlands

**Keywords:** gender difference and similarity, transient ischemic attack, stroke—diagnosis, telephone triage, help-seeking, sex differences and similarities

## Abstract

**Background:** In the Netherlands, a digital decision support system for telephone triage at out-of-hours services in primary care (OHS-PC) is used. Differences in help-seeking behavior between men and women when transient ischemic attack (TIA) or stroke is suspected could potentially affect telephone triage and allocation of urgency.

**Aim:** To assess patient and call characteristics and allocated urgencies between women and men who contacted OHS-PC with suspected TIA/stroke.

**Methods:** A cross-sectional study of 1,266 telephone triage recordings of subjects with suspected neurological symptoms calling the OHS-PC between 2014 and 2016. The allocated urgencies were derived from the electronic medical records of the OHS-PC and the final diagnosis from the patient's own general practitioner, including diagnoses based on hospital specialist letters.

**Results:** Five hundred forty-six men (mean age = 67.3 ± 17.1) and 720 women (mean age = 69.6 ± 19.5) were included. TIA/stroke was diagnosed in 294 men (54%) (mean age = 72.3 ± 13.6) and 366 women (51%) (mean age = 78.0 ± 13.8). In both genders, FAST (face-arm-speech test) symptoms were common in TIA/stroke (men 78%, women 82%) but also in no TIA/stroke (men 63%, women 62%). Men with TIA/stroke had shorter call durations than men without TIA/stroke (7.10 vs. 8.20 min, *p* = 0.001), whereas in women this difference was smaller and not significant (7.41 vs. 7.56 min, *p* = 0.41). Both genders were allocated high urgency in 75% of the final TIA/stroke cases.

**Conclusion:** Overall, patient and call characteristics are mostly comparable between men and women, and these only modestly assist in identifying TIA/stroke. There were no gender differences in allocated urgencies after telephone triage in patients with TIA/stroke.

## Introduction

Early recognition and timely diagnosis of patients with a transient ischemic attack (TIA) or stroke are vital to initiate interventions that can improve outcome and prevent recurrent stroke (1–3). The adage “time is brain” is often used in this context. Prompt action is initially dependent on the recognition and help-seeking behavior of the patient. A systematic review including 22 studies, mostly conducted in North America, showed better knowledge about potential signs and symptoms of stroke in women than men (4). Even among stroke survivors, women recognized traditional stroke warning signs more often than men (5). Despite this, women do not arrive faster at the hospital compared to men (6). There are even studies that have reported that women are more likely to delay seeking help compared to men (7, 8). A possible role in these delays is personality traits, such as “high anxiety,” in the decision process. Furthermore, a strong association has been reported between advice by others to seek help and shorter reaction times emphasizing the importance of intervention by family members or significant others (7). Women with a stroke, however, are more likely to live alone in their homes or in institutions, likely due to the fact that women tend to have stroke at older ages and generally outlive their partners (9).

In the Netherlands, the majority of patients with suspected TIA or stroke contact the general practitioner (GP) first (10). During evenings, nights, and weekends, such care is provided by the out-of-hours services in primary care (OHS-PC). Similar to other European countries, Australia, and New Zealand, the initial contact is by telephone, and trained nurses perform triage while supervised by GPs (11–13). The aims of standardized telephone triage are to assess the severity of patients' complaints and to allocate adequate urgencies based on a decision tree. These automatically generated urgencies are linked to a certain time period within which a GP or ambulance should examine the patient. The initial triage needs to differentiate urgencies based on the presence of symptoms possibly related to TIA/stroke. Differences in help-seeking behavior between women and men when TIA/stroke is suspected could potentially influence the telephone triage. When this results in different urgency allocations by gender, it could ultimately influence outcome. We therefore aimed to investigate differences in patient and call characteristics between women and men who contacted OHS-PC with suspected TIA/stroke and evaluate which factors were related to the final diagnosis TIA/stroke respectively in men and women.

## Methods

### Design and Setting

We conducted a cross-sectional study in which we analyzed real-life telephone triage recordings of nine OHS-PC locations in the vicinity of Utrecht, the Netherlands, between 2014 and 2016. These OHS-PCs provide out-of-hours primary care for ~1.5 million people, handling 400,000 triage calls per year.

We evaluated patients with symptoms suggestive of TIA or stroke. The triage recordings were selected in a two-step inclusion procedure, i.e., (i) selection based on the International Classification of Primary Care (ICPC) codes that are linked to the call and reflected our study domain (i.e., K89, K90, N17, N18, N19, N29, N89, N91) and (ii) keywords in the OHS-PC electronic medical records suggesting TIA/stroke (e.g., neurological deficit, arm or leg weakness, face drooping, communication problem, visual problems, sensory disturbances, and common synonyms) (14). Both patients with ongoing and already faded symptoms were included. A detailed description of the ICPC codes, medical keywords, and inclusion and exclusion criteria has been published elsewhere (15). We selected and relistened a random sample of 1,953 calls, and in 1,266 of these, we were able to retrieve the final diagnosis *via* the patient's GP (16).

### Variables

For data collection, we used two sources: the triage recordings and the electronic health records (EHRs) of the OHS-PC. Two researchers and 15 medical students listened to the triage recordings while blinded to the final outcome and collected data on signs and symptoms, medical history, and assigned urgencies using a standardized case report form. Additional patient and call characteristics were retrieved from the EHR of the OHS-PC. Autonomous nervous system (ANS)–related symptoms were defined as any of following: nausea, sweating, and pallor/ashen skin. Atypical symptoms were one or more of the following: consciousness decreased or lost, lightheadedness or fainting, dizziness/vertigo, weakness of both arms and/or both legs, sensory disturbances in both arms and/or both legs, blurry vision, and shortness of breath.

### TIA/Stroke

The final diagnosis of each patient who has contacted the OHS-PC was retrieved from the EHR of the patient's own GP. Thus, for all patients referred to the hospital (71%), the final diagnosis of TIA or stroke was based on discharge letters from the neurologist (or the emergency department). For patients who were not referred to the hospital, we used follow-up data from the health records of the OHS-PC and GPs for up to 1 month to capture a final diagnosis.

### Urgency Allocation

Triage nurses at Dutch OHS-PCs use a five-level, semiautomatic decision support tool called the “Netherlands Triage Standard” (NTS)^1^ which is partly derived from the physically oriented Manchester Triage System (17). After the initial ABCD (airway, breathing, circulation, and disability) check, triage nurses have to choose the most appropriate of 56 “main complaints” based on the patient's initial symptom presentation in order to start with the underlying algorithm of the NTS. After completing a few key questions and filling out the patient's answers in the NTS, an urgency level is automatically generated, which is linked to the maximum response time by which medical help should be provided (17). These urgency levels are U1 (ambulance dispatch within 15 min), U2 (GP consultation within 1 h), U3 (GP consultation within 3 h), U4 (GP consultation within 24 h), and U5 (telephone advice) (NTS). GP consultation consists of either a home visit or an appointment at the OHS-PC. The NTS urgency can be overruled by the triage nurse, which is mostly done only after first consulting a supervising GP (11). Urgency was dichotomized as high (U1 or U2) and low (U3, U4, or U5) urgency.

### Ethical Approval

The Medical Ethics Review Committee Utrecht approved this study (National Trial Register identification no.: Trial NL7134).

### Statistics

Men with TIA/stroke were compared with men with no TIA/stroke and similar for women. We used the χ^2^ test for categorical variables and the independent-samples *t*-test for continuous variables. We took multiple comparisons in account by controlling the false discovery rate (18). In addition, the final diagnoses were compared by men and women with age-adjusted general linear models to obtain man-to-woman differences (M–W Δ) and 95% confidence intervals (CIs). Lastly, as high-urgency allocation is especially crucial in major stroke, we analyzed the distribution of allocated urgency (low vs. high) in men and women with no TIA/stroke, minor stroke, and major stroke.

All analyses were done with the use of SPSS (version 21; SPSS, Chicago, IL, USA), and associations were judged to be significant with *P* < 0.05.

## Results

### Cohort Characteristics

Table 1 describes the patient and call characteristics of all subjects. We included 546 men (43%) and 720 (57%) women suspected of TIA/stroke in our cohort. The average age was 68.6 years [standard deviation (SD) ±18.5 years]. The face-arm-speech test [FAST; (1≥) face dropping, arm weakness, speech problem] was positive in 72% and atypical symptoms were reported in 42% of the patients. The caller was the patient himself or herself in about a quarter of the cases. Overall, the gender of the caller was in one third man. The vast majority (91%) of patients still had symptoms when they called OHS-PC (90% men vs. 92% women), and at the moment of contact, 46% had short-lasting symptoms <4.5 h. The average duration of the call was 7 min and 44 s (±3:44). A high urgency (U1 or U2) was allocated in 68%.

**Table 1:** Patient and call characteristics by gender of subjects with suspected TIA/stroke.

	**All patients**	**Men**	**Women**
	**(*n* = 1,266)**	**(*n* = 546)**	**(*n* = 720)**
**Patient characteristics**			
Age patient	68.6 ± 18.5	67.3 ± 17.1	69.6 ± 19.5
**Medical history**			
TIA or stroke	346 (27%)	163 (30%)	183 (25%)
Cardiovascular	677 (54%)	314 (58%)	363 (50%)
Use of antithrombotics	438 (35%)	213 (39%)	225 (31%)
**Mentioned symptoms**			
Face dropping	351 (28%)	153 (28%)	198 (28%)
Arm weakness	329 (26%)	134 (25%)	195 (27%)
Speaking problems	598 (47%)	258 (47%)	340 (47%)
Positive FAST test	905 (72%)	386 (71%)	519 (72%)
Leg weakness	284 (22%)	136 (25%)	148 (21%)
ANS symptoms	290 (23%)	101 (19%)	189 (26%)
Atypical/non-focal	529 (42%)	204 (37%)	325 (45%)
Headache	286 (23%)	108 (20%)	178 (25%)
**Call characteristics**			
Gender caller, man	409 (32%)	196 (36%)	213 (30%)
Patients calls	306 (24%)	137 (25%)	169 (24%)
Relation calls	693 (55%)	331 (61%)	362 (50%)
Partner	344 (27%)	210 (38%)	134 (19%)
Family member	319 (25%)	115 (21%)	204 (28%)
Neighbor	30 (2%)	6 (1%)	24 (3%)
Professional/other calls	267 (21%)	78 (14%)	189 (26%)
Duration call	07:44 ± 03:44	07:38 ± 03:34	07:48 ± 03:52
GP actively consulted	165 (13%)	62 (11%)	103 (14%)
Concern mentioned	580 (46%)	255 (47%)	325 (45%)
**Timing of call**			
Still symptoms	1,151 (91%)	489 (90%)	662 (92%)
No longer symptoms	100 (9%)	49 (9%)	51 (7%)
Unknown	15 (1%)	8 (2%)	7 (1%)
**Duration symptoms**			
Overall <4.5 h	581 (46%)	240 (44%)	341 (47%)
Overall >4.5 h	402 (32%)	204 (37%)	198 (28%)
**Allocated urgency**			
High urgency (U1 or U2)	865 (68%)	375 (69%)	490 (68%)

*Data are presented as number (%) or mean ± SD*.

### TIA/Stroke vs. No TIA/Stroke

In 660 patients (52%) who contacted the OHS-PC, the final diagnosis was a TIA or stroke. Table 2 describes all the final diagnoses by gender. In 294 men (54%) and 366 women (51%), the final diagnosis was TIA/stroke [age-adjusted M–W Δ 5.5% (95% CI = 0.4–10.6)]. Men more often had a TIA (26%) compared to women [21%; age-adjusted M–W Δ 5.5% (95% CI = 0.9–10.1)], whereas men less often received the diagnosis migraine (2%) compared to women [5%; age-adjusted M–W Δ −3.7% (95% CI = −5.6 to −1.8)]. The other diagnoses were comparable by gender.

**Table 2:** Diagnosis by gender of patients with suspected TIA/stroke.

	**Men (M)**	**Women (W)**	**M–W Δ**
	**(*n* = 546)**	**(*n* = 720)**	**age adj**.
**TIA/stroke**	**294 (54%)**	**366 (51%)**	5.5% (0.4, 10.6)^*^
TIA	139 (26%)	152 (21%)	5.5% (0.9, 10.1)^*^
Minor stroke	65 (12%)	78 (11%)	1.5% (−2.1, 5.0)
Major ischemic stroke	78 (14%)	121 (17%)	−1.7% (−5.7, 2.3)
Hemorrhagic stroke	11 (2%)	14 (2%)	0.2% (−1.3, 1.8)
Subarachnoid hemorrhagic	1 (<1%)	1 (<1%)	n.a.
**Mimics**	**252 (48%)**	**354 (49%)**	1.6% (−2.0, 5.3)
Epileptic seizure	9 (2%)	14 (2%)	−0.3% (−1.8, 1.2)
Facial palsy	60 (11%)	62 (9%)	1.1% (−2.0, 4.2)
Migraine	8 (2%)	33 (5%)	−3.7% (−5.6–1.8)^*^
Peripheral vestibular	23 (4%)	41 (6%)	−1.7% (−4.2, 0.7)
Psychogenic	22 (4%)	29 (4%)	−0.4% (−2.6, 1.7)
Syncope	12 (2%)	18 (3%)	−0.2% (−1.9, 1.5)
Other	118 (22%)	157 (22%)	−0.3% (−4.9, 4.3)

*M–W Δ age adj. = man-to-woman age-adjusted difference*.

* *Statistically significant (within 95% CI = p < 0.05)*.

Table 3 describes the patient and call characteristics for men and women separated by TIA/stroke and no TIA/stroke diagnosis. In both genders, patients with TIA/stroke were older and more often diagnosed in the hospital compared to patients with no TIA/stroke. Men with TIA/stroke less often had a TIA or stroke in their medical history (36%) than those with no TIA/stroke (50%, *p* < 0.001), whereas in women the opposite was observed (30 vs. 20%, *p* = 0.002). In both genders, the frequency of mentioned typical symptoms (e.g., FAST) was more often mentioned by the patient with TIA/stroke; however, they were also frequent in patients with no TIA/stroke and *vice versa* for less classical symptoms, such as headache, ANS symptoms, and atypical symptoms.

**Table 3:** Patient and call characteristic comparison by gender of subjects with and without TIA/stroke.

	**Men**			**Women**		
	**Stroke/TIA**	**No stroke/TIA**	***p***	**Stroke/TIA**	**No stroke/TIA**	***p***
	***n* = 294**	***n* = 252**		***n* = 366**	***n* = 354**	
**Diagnosed by**						
GP/OHS PCS	36 (12%)	81 (32%)	< 0.001	68 (19%)	119 (62%)	< 0.001
Neurology/ED	255 (87%)	144 (57%)	< 0.001	296 (81%)	209 (59%)	< 0.001
Other/unknown	3 (1%)	27 (11%)	< 0.001	4 (1%)	26 (7%)	< 0.001
**Patient characteristics**						
Age patient, years	72.3 ± 13.6	61.3 ± 18.9	< 0.001	78.0 ± 13.8	61.0 ± 20.7	< 0.001
**Medical history**						
TIA or stroke	107 (36%)	125 (50%)	< 0.001	111 (30%)	72 (20%)	0.002
Cardiovascular	187 (64%)	127 (50%)	0.002	212 (58%)	151 (43%)	< 0.001
Antithrombotic use	131 (45%)	82 (33%)	0.004	153 (42%)	72 (20%)	< 0.001
**Symptoms mentioned**						
Face dropping	78 (27%)	75 (30%)	0.40	96 (26%)	102 (29%)	0.44
Arm weakness	91 (31%)	43 (17%)	< 0.001	137 (37%)	58 (16%)	< 0.001
Speaking problems	174 (59%)	84 (33%)	< 0.001	218 (60%)	122 (34%)	< 0.001
Positive FAST test	228 (78%)	158 (63%)	< 0.001	300 (82%)	219 (62%)	< 0.001
Leg weakness	86 (29%)	50 (20%)	0.01	96 (26%)	52 (15%)	< 0.001
Atypical/non-focal	92 (31%)	112 (44%)	0.02	149 (41%)	176 (50%)	0.02
ANS symptoms	42 (14%)	59 (23%)	0.01	68 (19%)	121 (34%)	< 0.001
Headache	47 (16%)	61 (24%)	0.02	65 (18%)	113 (32%)	< 0.001
**Call characteristics**						
Gender caller, man	85 (29%)	111 (44%)	< 0.001	101 (28%)	112 (32%)	0.24
Patients calls	53 (18%)	84 (33%)	< 0.001	52 (14%)	117 (33%)	< 0.001
Partner calls	120 (41%)	90 (36%)	0.22	51 (14%)	83 (23%)	0.001
Family/neighbor calls	65 (22%)	56 (22%)	0.23	137 (37%)	91 (26%)	0.55
Professional/other calls	56 (19%)	22 (9%)	0.001	126 (34%)	63 (18%)	< 0.001
Duration call	07:10 ± 3:11	08:20 ± 3:53	0.001	07:41 ± 3:50	07:56 ± 3:54	0.41
GP was actively consulted	26 (9%)	36 (14%)	0.046^*^	47 (13%)	56 (16%)	0.25
Caller mentioned concern	135 (46%)	120 (48%)	0.69	157 (43%)	168 (47%)	0.22
**Timing**						
Still symptoms	258 (88%)	231 (92%)	0.14	336 (92%)	326 (92%)	0.89
<4.5 h	124 (42%)	83 (33%)		170 (46%)	132 (37%)	
≥4.5 h	90 (31%)	99 (39%)		83 (23%)	109 (31%)	
Duration unknown	44 (15%)	49 (19%)		83 (23%)	85 (24%)	
No longer symptoms	31 (11%)	18 (7%)	0.17	25 (7%)	26 (7%)	0.79
Unknown	5 (2%)	3 (1%)	0.62	5 (1%)	2 (1%)	0.27
**Duration symptoms**						
Overall <4.5h	144 (49%)	96 (38%)	0.01	192 (53%)	149 (42%)	0.01
Overall>4.5h	100 (34%)	104 (41%)	0.08	86 (24%)	112 (32%)	0.01
Unknown	50 (17%)	52 (21%)	0.28	88 (24%)	93 (26%)	< 0.001

*Data are presented as n (%) or means ± SD*.

*Gender differences between TIA/stroke and no TIA/stroke were calculated with χ^2^ or T-test*.

* *Statistical significance lost after correction for false discovery rate*.

The caller in men with TIA/stroke was most often the partner, also in men with no TIA/stroke. In women with TIA/stroke, a family member or neighbor was most often the caller, whereas in women with no TIA/stroke, the patients themselves were most often the caller. The duration of the call was shorter in men with TIA/stroke (07:10 ± 3:11) compared to men with no TIA/stroke (08:20 ± 3:53, *p* = 0.001). The GP was less often actively involved in the phone call with men with TIA/stroke (9%) compared to men with no TIA/stroke (14%, *p* = 0.046). In contrast to men, the duration of the call is comparable between women with TIA/stroke (07:41 ± 3:50) and women with no TIA/stroke (07:56 ± 3:54, *p* = 0.41). Also, consultation of a GP by the triage nurse was comparable between women with TIA/stroke (13%) compared to women with no TIA/stroke (16%, *p* = 0.25).

The majority of men and women still had symptoms when they contacted the OHS-PC, both in patients with TIA/stroke and in patients with no TIA/stroke. The majority of these patients reported a symptom duration <4.5 h, and the proportion was higher in both genders with TIA/stroke (men 42%, women 46%) compared to no TIA/stroke (men 33%, women 37%). High urgency was allocated slightly more often in the TIA/stroke diagnosis group compared to no TIA/stroke in both genders.

### Urgency Allocation by Gender

**Figure 1:**
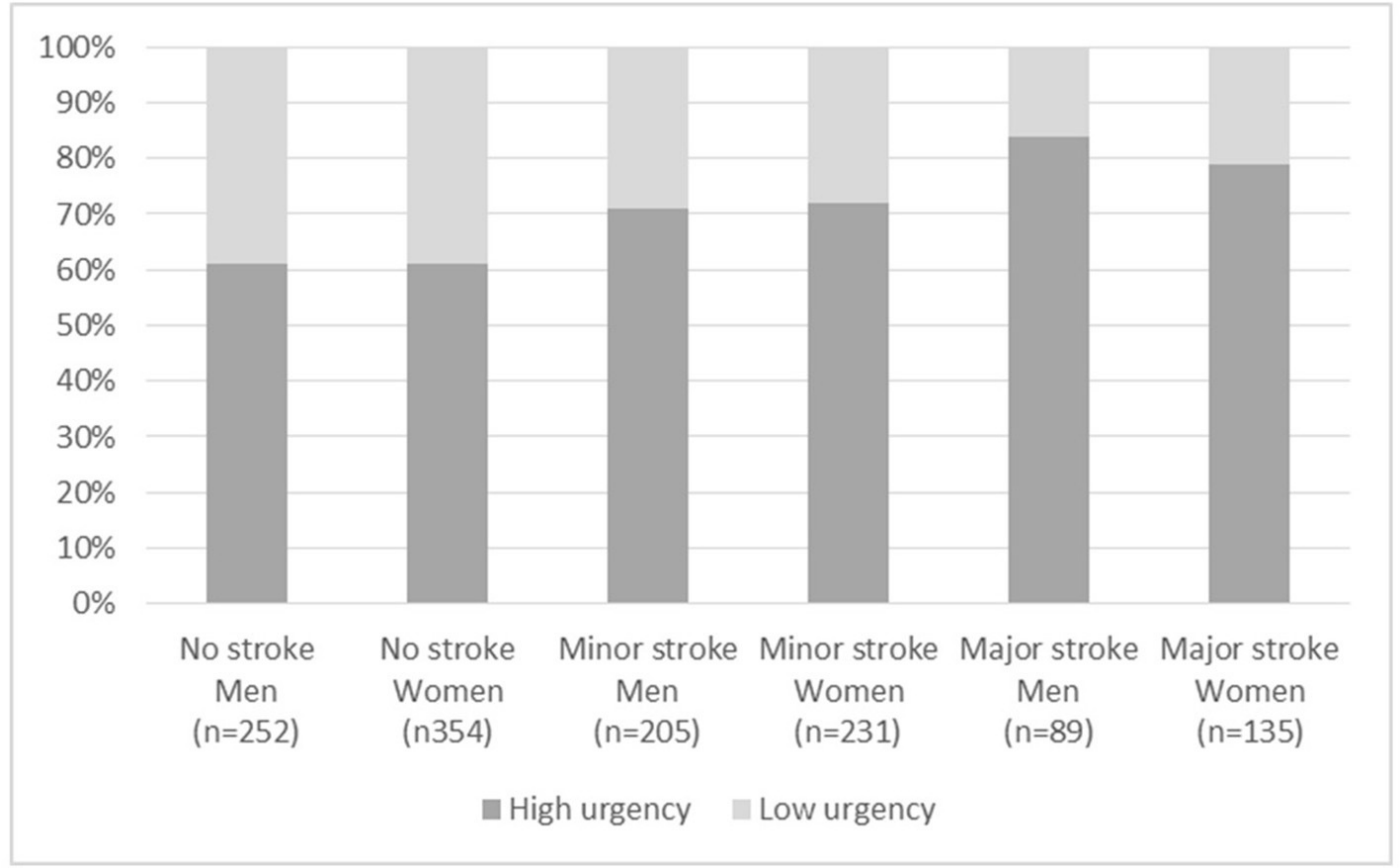
Allocated urgency by clinical diagnosis in women and men. Data are presented as *n* (%) and statistical differences were calculated with χ^2^ test. U1–2, NTS level of urgency; 1 = life threatening; 2 = emergent. U3–5, NTS level of urgency; 3 = urgent, 4 = nonurgent, 5 = advice.

The distribution of allocated urgency (low vs. high) in men and women with no TIA/stroke, minor stroke, and major stroke is shown in Figure 1. Both men (84%) and women (79%) with major stroke had the highest percentage of high-urgency allocation. This small difference did not reach statistical significance. In men with minor stroke, high urgency was allocated in 71%, and in women in 72%. High-urgency allocation was also common in patients with no TIA/stroke in both genders (61%).

## Discussion

Our results showed that most differences in call and patient characteristics between patients with and without TIA/stroke are comparable between men and women. There are some differences in risk factors and call characteristics regarding caller and duration of the call. High urgencies are allocated frequently in both men and women with suspected TIA/stroke that contact the OHS-PS. In both genders, a high urgency is more often allocated in patients with than in patients without TIA/stroke.

Patient characteristics between patients with and without TIA/stroke were comparable between men and women. For both genders, the typical symptoms (FAST) were more often mentioned by patients with TIA/stroke; however, they were also frequent in patients with no TIA/stroke. And *vice versa* for less classical symptoms, such as headache, ANS, and nonfocal symptoms. Discriminating between TIA/stroke or no TIA/stroke based on anamnestic symptoms only is challenging (19, 20). Neurological deficits may also be caused by multiple other (less urgent) disorders. Not surprisingly, the NTS decision support tool used in Dutch OHS-PC performs moderately regarding sensitivity and specificity in allocating adequate urgencies to patients with and without TIA/stroke (16). Literature on this topic is scarce. A German prospective cohort study evaluated a comparable triage system for patients suspected of neurological disease (not otherwise specified), however, at the emergency department (21). The prevalence of severe cerebrovascular events is higher in the emergency department compared to primary care, which could be reflected by more evident clinical presentations. In this emergency department study, 391 patients from 543 (72%) with neurological symptoms were triaged as to be seen by a physician within an hour (21). Our study extends these previous findings by showing that, in case of suspected TIA/stroke, the OHS-PC allocates high urgencies (seen within an hour) in 65%, and this is similar in men and women. This is in line with previous findings for acute coronary syndrome (ACS). Discriminating patients with and without ACS in case of complaints of chest discomfort is equally difficult for men and women who contacted OHS-PC (22). Both genders with chest discomfort received high-urgency allocation in ~65% and in women with ACS in 96%, and in men in 88% (22).

There were some gender differences in call characteristics between patients with and without TIA/stroke. The caller on the phone differed. In men with and without TIA/stroke, most often their partner is on the phone, whereas in women with TIA/stroke, another family member or neighbor calls, and in women with no TIA/stroke, the patients themselves call most often. Previous studies have shown that prior to stroke, women are more likely to live alone in their homes or in institutions, likely due to the fact that women tend to have stroke at older ages and generally outlive their partners (9). In our cohort, men with stroke (72.3 ± 13.6) were on average 6 years younger than women with stroke (78.0 ± 13.8, *p* < 0.001). The gender of the caller overall was most often woman (68%). This is consistent with previous studies (13, 23). Another difference in call characteristics was the mean call duration. Interestingly, men with TIA/stroke had significant shorter telephone call durations than men without TIA/stroke (7.10 vs. 8.20 min, *p* = 0.001), whereas in women this difference was smaller and not significant (7.41 vs. 7.56 min, *p* = 0.41), suggesting that triage nurses were prompted with an urgency sooner in men with TIA/stroke. In previous studies on gender differences in OHS-PS call duration, an opposite finding was presented for ACS. The mean call duration was longer in women with no ACS compared to women with ACS, whereas there was no difference in duration for men (22). Our finding is therefore not general for one gender but seems dependent on the clinical diagnosis that is suspected. It could be postulated that there are symptoms or clues in the telephone contact about male patients that are recognized better by the triage nurse or GP. However, as most characteristics have a similar pattern in TIA/stroke and no TIA/stroke in both genders in our study, these clues might be difficult to quantify and more “between the lines.” Moreover, perhaps the caller in male patients (most often the female partner) is better able to apprise and communicate the urgency. The aforementioned study on ACS did not report on the (gender of the) caller. It has previously been shown that patients with TIA/stroke, living with someone, or being married improved time to arrival in men only (24). In general, seeking professional health care advice and performing care for others are linked with ideal femininity (23). Correspondingly, men can enact masculinity through refraining from seeking health care, in this case by refraining from calling.

A timely diagnosis of patients with a TIA or stroke is vital to initiate interventions that can improve the outcome. Especially, in ischemic stroke, the sooner treatment is initiated, the more benefit is derived (25). However, when the symptoms are still present at the moment of contact with the OHS-PC, TIA cannot be discriminated from stroke. In the current study, we showed that the urgency allocation for TIA/stroke is similar for women and men. Previous studies on gender differences in prehospital delay for symptoms of stroke are heterogenic in design, hampering pooled comparison and general conclusions [review (6)]. Overall, many studies report comparable delays for women and men; however, some studies (7, 8) indicate that women experience a longer time delay than do men. In the current study, the far majority of both genders contacted the OHS-PC while they still had symptoms with a duration <4.5 h, which is within the time window of treatment.

Improvement of the current telephone triage for the identification of TIA/stroke in both women and men with symptoms suggestive of acute cerebrovascular disease is warranted. Currently, the NTS algorithm “neurological deficit” (i.e., 1 of the 56 “main complaints”) does not include inquiring after well-established risk factors, while this would likely increase accuracy of allocated urgency. The current study showed many similarities in call and patient characteristics between men and women; however, there were also some differences, especially in well-established risk factors such as age and a history of TIA/stroke. Women with TIA/stroke were, on average, 6 years older than men. At a young age, the risk of stroke in women is lower compared to men; yet, once women pass menopause, their incidence rates of stroke are far greater than rates in men (26). Furthermore, in the current study, a TIA or stroke in the medical history was more common (50%) in men with no TIA/stroke than men with TIA/stroke (36%). On the contrary, TIA or stroke in the medical history was more common (30%) in female patients with TIA/stroke compared to female patients with no TIA/stroke (20%). This is in line with previous literature, showing that women are at higher risk of recurrent stroke, partially explained by women's greater life expectancy (26). Incorporating risk factors while taking into account the gender of the patient might improve the NTS triage. Furthermore, 45% of the women reported atypical symptoms, and 37% of the men, but both genders more often had atypical symptoms when the final diagnosis was no TIA/stroke compared to TIA/stroke. It has been postulated that women more frequently present with nonconventional symptoms of cerebrovascular pathology, which can delay the diagnosis; however, the available literature is heterogeneous (27). The issue of non-focal cerebrovascular pathology could not be well-covered in our study, but seems an interesting future lead.

A strength of this study is the detailed telephone triage data from the OHS-PC completed with the final clinical diagnosis. Because the researchers were blinded to the final clinical outcome during data collection, the effect of hindsight bias was limited. In addition, we included nine OHS-PC locations covering both urban and rural areas of the Netherlands, which makes our findings generalizable not only to the whole of the Netherlands, but also to other countries with a similar health care system with a strong primary care that is organized in large-scale out-of-hours primary care centers, e.g., Scandinavian countries, Germany, Belgium, and the United Kingdom (28). A limitation was missing data on the final clinical outcome (35% of all relistened recordings). However, a previous detailed comparison in patient characteristics between those with a final diagnosis and those without showed that these groups were comparable (i.e., no indication of selection bias) (16). Therefore, we believe our results are generalizable to similar OHS-PC settings. Additionally, the final diagnosis was based on routine clinical care, rather than on a prespecified diagnostic evaluation. Furthermore, we had no information on the living situation (e.g., living with partner, alone, or assisted living), while it is possible that this could have influenced the recognition of symptoms and the treatment plan. A previous study has shown that men living alone or who are divorced are less likely to arrive at the hospital <3 h compared to men living with a partner or who are married, whereas this effect was not found in women (24). Furthermore, the GP might be more likely to plan a home visit for an elderly living alone.

## Conclusion

Discriminating patients with TIA or stroke from those without in patients with neurological deficits who contacted OHS-PC seems equally difficult for men and women. Despite that subtle gender-related differences are observed in triage of patients with complaints suspected for TIA/stroke, the allocated urgency is comparable for women and men.

## Data Availability Statement

The data analyzed in this study is subject to the following licenses/restrictions: The datasets generated during and/or analyzed during the current study are available from the corresponding author on reasonable request. Requests to access these datasets should be directed to D.Zwart@umcutrecht.nl.

## Ethics Statement

The Medical Ethics Review Committee Utrecht approved this study (National Trial Register identification number: Trial NL7134). Written informed consent for participation was not required for this study in accordance with the national legislation and the institutional requirements.

## Author Contributions

LE: conceptualization, methodology, software, formal analysis, writing—original draft, and visualization. SD: resources, methodology, and writing—review and editing. DE and KS: resources and writing—review and editing. FR: conceptualization, methodology, writing—review and editing, and funding acquisition. LK: conceptualization, methodology, and writing—review and editing. DZ: conceptualization, methodology, writing—review and editing, supervision, and funding acquisition. All authors contributed to the article and approved the submitted version.

## Conflict of Interest

The authors declare that the research was conducted in the absence of any commercial or financial relationships that could be construed as a potential conflict of interest.

## Footnotes

^1^NTS. Available online at: https://de-nts.nl/nts/basisprincipes-nts/.
